# Long-Term Alendronate Use Not without Consequences?

**DOI:** 10.1155/2009/253432

**Published:** 2010-01-27

**Authors:** M. P. Somford, G. F. A. E. Geurts, J. W. A. M. den Teuling, B. J. W. Thomassen, W. F. Draijer

**Affiliations:** ^1^Department of Orthopaedic Surgery, Academic Medical Center, Postbus 22660, 1100 DD Amsterdam, The Netherlands; ^2^Department of Orthopaedic Surgery, Orbis Medical Center, Postbus 5500, 6130 MB Sittard, The Netherlands; ^3^Department of Orthopaedic Surgery, Atrium Medical Center, Postbus 4446, 6401 CX Heerlen, The Netherlands; ^4^Department of Orthopaedic Surgery, Medical Centre Haaglanden, Postbus 432, 2501 CK The Hague, The Netherlands

## Abstract

A previously unknown side effect of biphosphonate use is emerging. In a specific patient group on long term biphosphonate therapy stress femur fractures seem to occur. The typical presentation consists of prodromal pain in the affected leg and/or a discrete cortical thickening on the lateral side of the femur in conventional radiological examination or the presentation with a spontaneous transverse subtrochanteric femur with typical features. We present three cases of this stress fracture in patients on bisphosphonate therapy. One of these patients suffered a bilateral femur fracture of the same type. In our opinion, in patients on bisphosphonate therapy who present with a spontaneous femur fracture, seizing therapy is advisable. In bilateral cases preventive nailing should be considered.

## 1. Introduction

Following earlier reports of spontaneous femur fracture in patients using alendronate, we present three cases with a similar course. All three of these patients presented with typical prodromal symptoms resulting in a specific fracture type discussed afterwards.

### 1.1. Case 1

Patient A, a 65-year-old woman, was seen at the emergency room with sudden pain in her right leg after getting up from a chair and an inability to bear weight. Her medical history showed rheumatoid arthritis since 2001 and hypertension. The medication list consisted of alendronate sodium 70 mg o.w. for four years, omeprazole 20 mg o.d., prednisone 10 mg o.d. for seven years, and metoprolol 100 mg o.d., plaquenil 200 mg t.d., etanercept 50 mg o.w.

**Figure 1:**
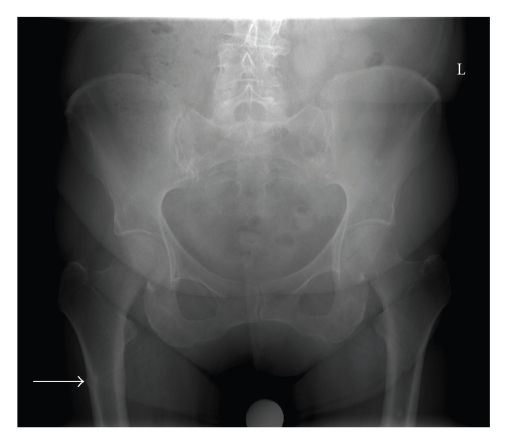
X-ray taken at first presentation of thigh pain on the right side. There is a sign of discrete lateral cortex thickening.

Four months prior to the patient's visit to the emergency room, the rheumatologist had consulted the neurologist and the orthopaedic surgeon because of persisting pain in the right leg and groin, but no neurological or orthopaedic explanation could be found. The only abnormality found was a thickening of the medial and lateral cortex of the right femur with a discrete local periostal reaction on the lateral cortex (Figure 1). Laboratory results were normal.

**Figure 2:**
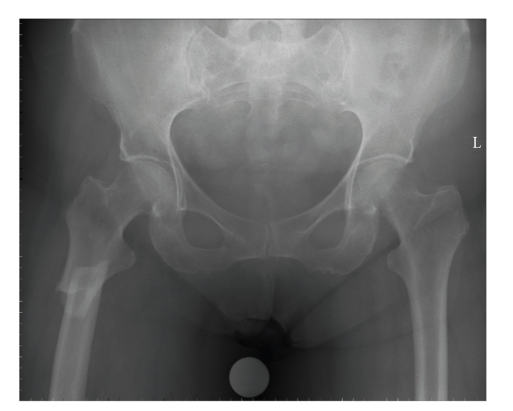
The subtrochanteric fracture through the lateral cortex thickening with a medial spike.

Radiological examination on admission showed a transverse subtrochanteric fracture of the right femur with a medial spike. The fracture was located at the same spot as the cortical thickening (Figure 2).

A closed reduction and internal fixation took place. The patient was discharged from the hospital in good condition a week later.

### 1.2. Case 2

Patient B, a 79-year-old woman, was seen at the emergency room because of a sudden inability to bear weight on her left leg. The patient related that she had heard a crack in her leg and had then fallen down. Her medical history showed osteoporosis and rheumatoid arthritis since 1987. The medication list consisted of prednisone 5 mg o.d. for 8 years, alendronic acid 70 mg o.w. for 12 years, calcium carbonate 500 mg o.d., methotrexate 20 mg o.w. for 7 years, furosemide 40 mg o.d., etoricoxib 60 mg o.d., potassium chloride 1200 mg o.d., lansoprazole 30 mg o.d., carvedilol 6.25 mg o.d., acenocoumarol, gliclazide 30 mg o.d., folic acid 10 mg o.w., and tramadole 100 mg o.d.

**Figure 3:**
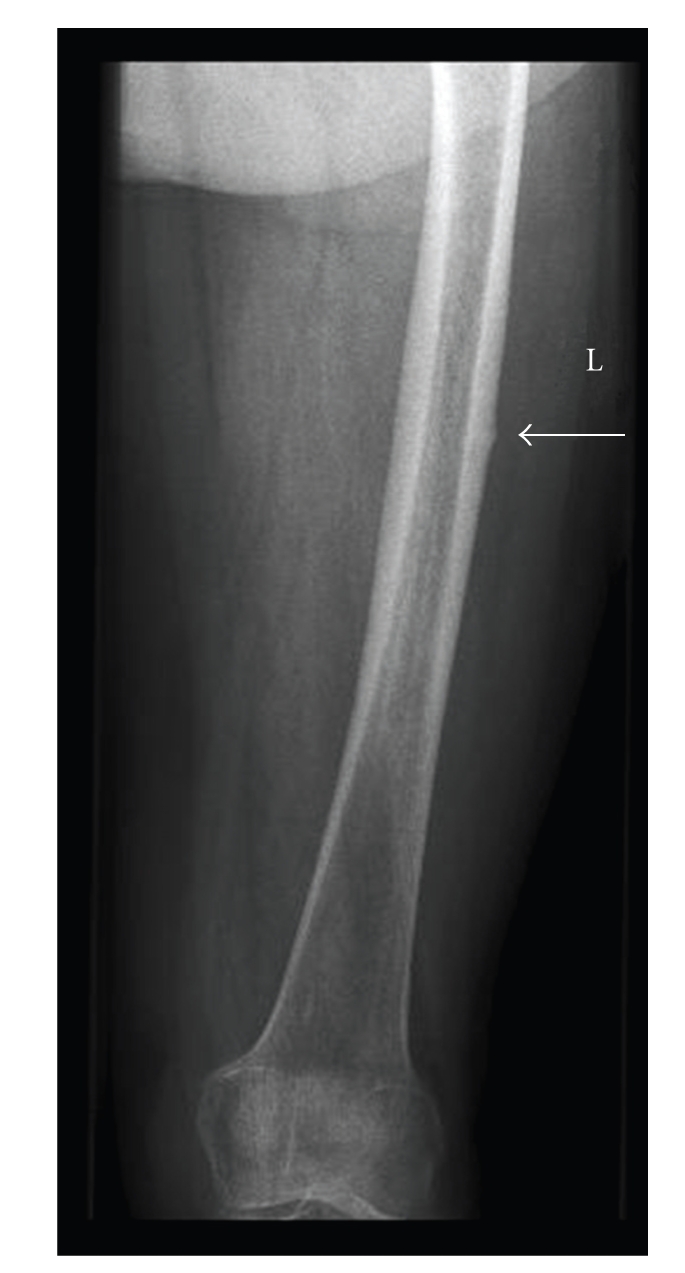
Cortical thickening (arrow) at the site of the pain in the left thigh.

Prior to the patient's visit to the emergency room, the rheumatologist had referred her to the orthopaedic surgeon because of pain in the left thigh. Radiological examination at that time showed a thickening of the medial and lateral cortex of the left femur with a discrete local periostal reaction on the lateral cortex (Figure 3). A bone scintigraphy showed a small focal lesion in the left femoral midshaft, and an additional computer tomography (CT) scan revealed a small thickening of the cortex at the level of the hotspot.

**Figure 4:**
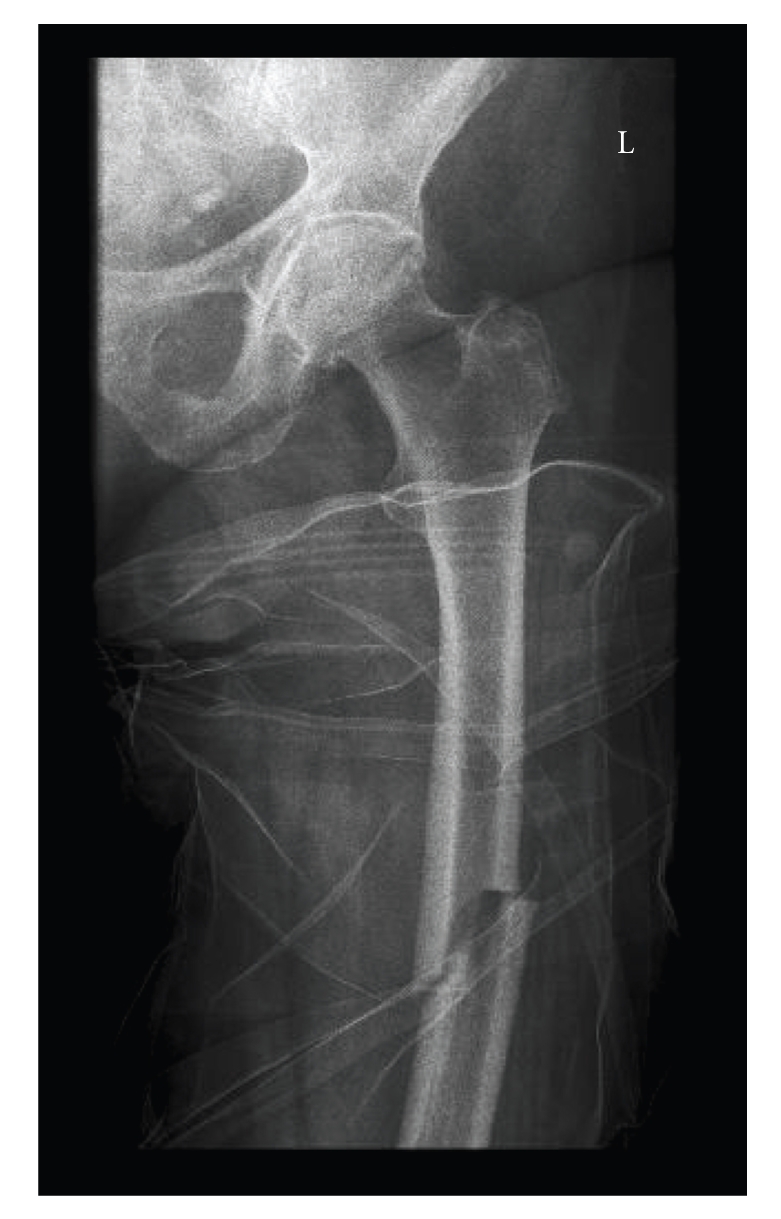
The subtrochanteric fracture through the thickening of the lateral cortex with a medial spike.

On physical examination in the emergency room, a diffuse swollen left thigh was noted with evident pain when stress was applied through the femur. Laboratory results were normal. Additional radiological examination showed a transverse femur shaft fracture (Figure 4). The fracture site was at the exact location of the cortical thickening.

Closed reduction and internal fixation took place. Bone collected after reaming of the femur was sent out for pathological examination. The pathological examination showed small fragments of cancellous bone tissue and bone marrow without signs of malignancy, the aspect of normal bone.

### 1.3. Case 3

Patient C, a 76-year-old woman, was admitted to hospital by the rheumatologist because of persisting pain in her left leg without a clear diagnosis. Her medical history showed rheumatoid arthritis since 1985. The medication list consisted of prednisone 5 mg o.d. for 9 years, omeprazole 20 mg o.d., alendronate sodium 70 mg o.w. for 12 years, methotrexate 15 mg o.w. for 8 years, folic acid 5 mg o.w. and infliximab infusion 200 mg once per 8 weeks for 3 years. 

The patient's complaints of leg pain had started approximately three months earlier without any preceding trauma. Since the start of the leg complaints, she had not been able to walk more than a few steps at a time. The neurologist was consulted but no neurological explanation was found. As blood samples, including hydroxyvitamin D, showed no abnormalities, the orthopaedic surgeon was consulted. 

**Figure 5:**
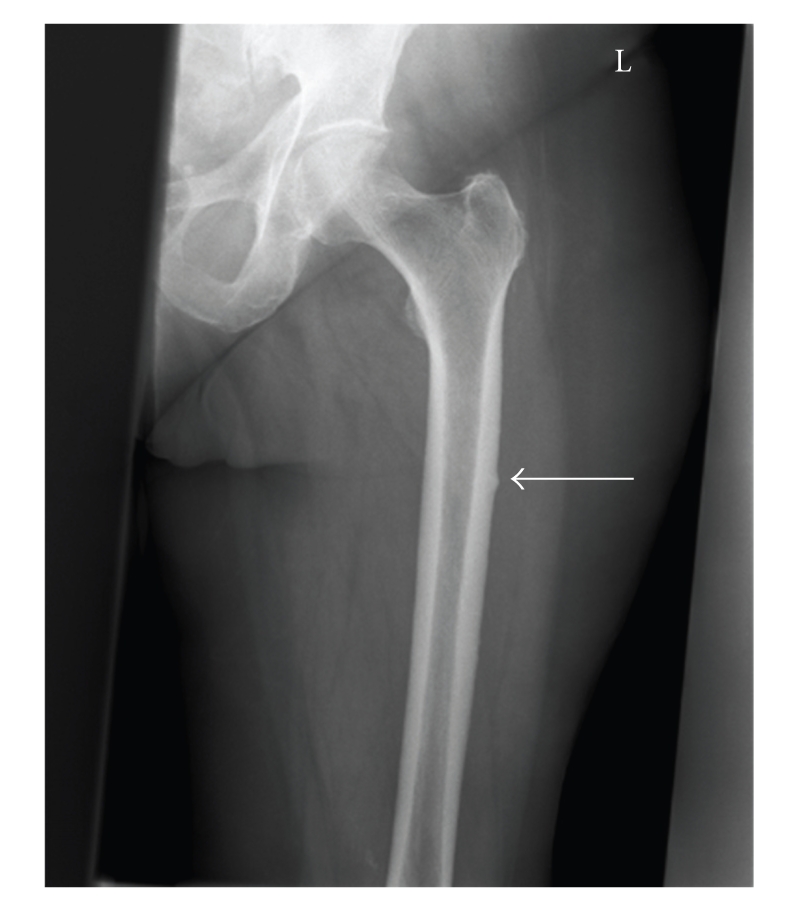
Thickening of the lateral cortex (arrow) at the site of the pain in the left thigh.

**Figure 6:**
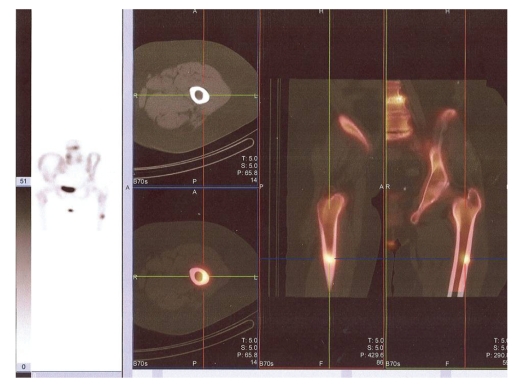
SPECT of the lesion on the left femur showing bilaterally thickened cortices and both internally and externally intact cortices. On the left side the bone scan showing a hotspot on the lateral side of the left femur.

Radiological examination of the pelvis and left upper-leg showed a thickening of the medial and lateral cortex with a discrete thickening of the cortex on the lateral side of the proximal femur (Figure 5). A bone scintigraphy showed a hotspot in the left femur. Single-photon emission computer tomography (SPECT) of this region also demonstrated a discrete thickening of the cortex without changes of the periost. The cortex was intact both internally and externally and showed increased thickness (Figure 6). An additional dual-energy X-ray absorptiometry (DEXA) scan of the lumbar vertebrae showed no signs of osteoporosis or osteopenia (T-score 0.1).

**Figure 7:**
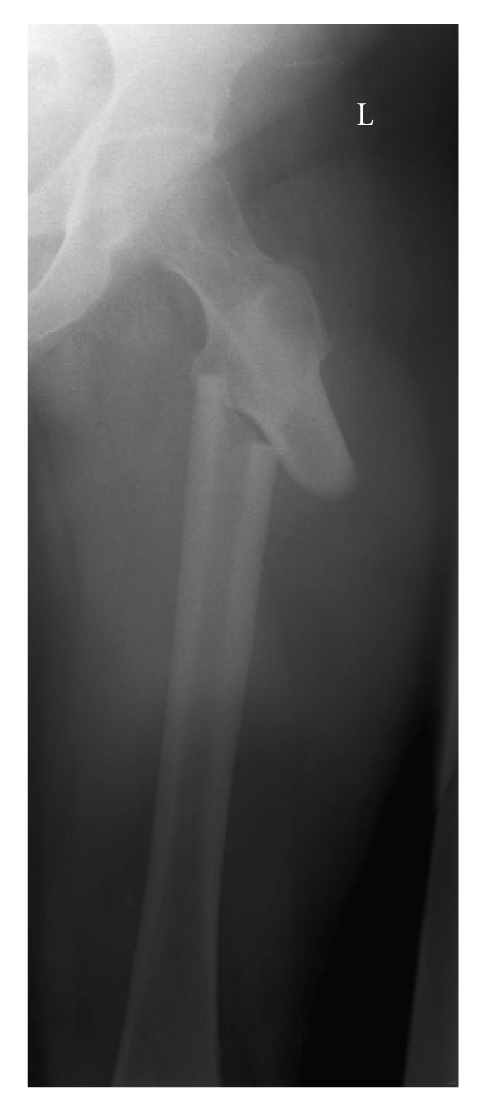
Fracture through the thickening of the lateral cortex with a medial spike.

Before any more tests could be performed, the patient heard a crack when she attempted to sit down on a chair. This turned out to be a transverse subtrochanteric fracture through the cortical thickening with a medial spike (Figure 7). Closed reduction and internal fixation took place.

A biopsy taken intraoperatively showed blood and fat with a normal haematopoiesis microscopically. Additional immunohistochemical investigation showed no signs of malignancy or osteoporosis. After the operation, the patient very quickly regained mobility and was free of pain.

**Figure 8:**
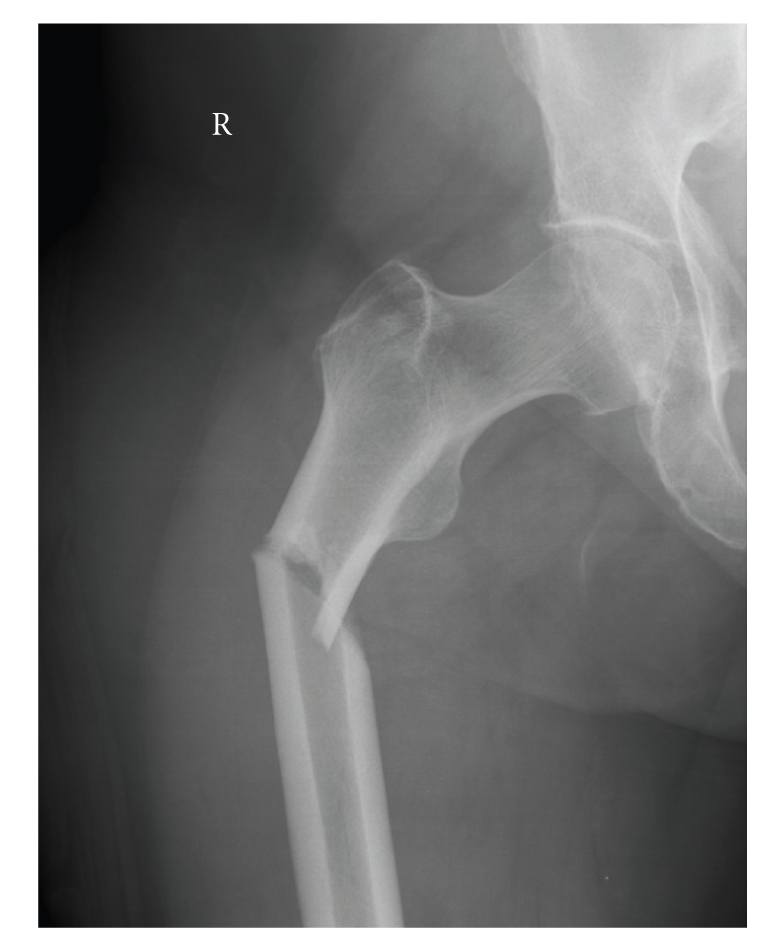
Subtrochanteric fracture in the same patient as Figures 5 and 6 on the contra-lateral side. The fracture has the exact same pattern.

The patient reappeared at the emergency room eight months later. She was using the same medication as before, except for the methotrexate. The patient had been suffering from pain in her right thigh for two weeks and had sustained a spontaneous subtrochanteric fracture of the right femur while walking (Figure 8). Upon examination it turned out that the patient had again suffered a transverse fracture at the exact location of a cortical thickening. A closed reduction and internal fixation took place. Postoperatively, the patient very quickly regained mobility and was discharged from the hospital after ten days.

## 2. Discussion

Since their introduction in The Netherlands in 1996, bisphosphonates are often prescribed for the prevention and treatment of osteoporosis. General practitioner's guidelines mention it along with calcium and vitamin D as the preferred preventive medical treatment [1]. One of the guidelines' core messages is that patients with long-term use of corticosteroids as well as patients with osteoporosis could be treated with bisphosphonates (alendronate and risendronate). Bisphosphonates act as bone resorption inhibitors in healthy individuals as well as in individuals with increased resorption, in whom they can restore the balance [2]. 

The guidelines advise against prescribing bisphosphonates for longer than five years as data on the long-term effects of bisphosphonates are lacking. However, they do recommend continuing the use of bisphosphonates in patients with long-term use of corticosteroids. In these patients, the bisphosphonates should only be discontinued at the same time as the corticosteroids [1].

Recently, several reports have warned for the possible negative effects of long-term alendronate use. These reports associate long-term alendronate use with subtrochanteric fractures. It has been known for a long time that the subtrochanteric region is subjected to maximal bending stress and is, as such, the strongest part of the femur [3].

Consequently, a fracture in this region following low-energy trauma seems unlikely. Only 10–34% of all proximal femur fractures occur in the subtrochanteric region [4].

In a retrospective study, Goh et al. describe a group of 13 Asian female patients who presented with low-energy subtrochanteric fractures [5]. Nine patients were taking alendronate. There was a clear difference in age and fracture type between patients who used alendronate and patients who did not. Those using alendronate mainly presented with a simple transverse fracture at a mean age of 66.9 years. The other patients presented with more comminutive fractures and had an average age of 80.3 years. The average use of alendronate was 4.2 years (range 2.5–5 years). Remarkably, five of the women who used alendronate reported experiencing pain in the fractured limb two to six months prior to the injury. Patients without alendronate use did not experience these prodromal symptoms. 

Neviaser et al. collected data retrospectively on 70 low-energy femoral shaft fractures [6]. In this group, 25 female patients (23 Caucasian and 2 Asian) used alendronate. The average period that alendronate was used was 6.2 years (range 1–10 years). Data analysis showed that 76% of the patients using alendronate had a specific fracture pattern, which was seen in only 2% of the patients without alendronate use. This fracture pattern consists of a simple transverse fracture of the femur with a unicortical spike in an area of hypertrophy. Furthermore, it was calculated that such a fracture pattern is 98% specific for alendronate-using patients. It is worth noting that in the alendronate group the patients that did display the fracture pattern had been using alendronate significantly longer (6.9 years versus 2.5 years for short-term users).

The same specific fracture pattern is also described by Kwek et al. [7]. They carried out a retrospective study on 17 Asian female patients with low-energy trauma subtrochanteric fractures. All patients had received alendronate therapy for a mean of 4.4 years (range of 2–8 years). The average age of the patients was 66 years (range of 53–82 years). Prodromal symptoms were present in 13 patients (76%). Six patients had a stress zone in the subtrochanteric region of the contra-lateral femur. Four patients sustained bilateral subtrochanteric fractures. The affected region might just be due to the maximal bending stress of the subtrochanteric region, especially the lateral cortex [3].

In the three cases related above, the patients are all female and are respectively 65, 76, and 79 years of age. They are all long-term alendronate users. In our group an average use of 10 years (range of 4–13 years) is present. Following the Dutch general practitioner's guidelines, they should use alendronate for an extended period as long as they are using corticosteroids too. All three patients showed the typical prodromal symptoms and a thickening of the lateral cortex that resulted in subtrochanteric fractures. The fractures were not caused by any trauma and might be described as spontaneous fractures. But in our opinion the thickening of the cortex prior to the fracturing of the femur makes it a stress or fatigue fracture. This evolving of a cortical thickening into a (fatigue) fracture is particularly clear in our third case. The fracture patterns itself accorded with the typical pattern described by Kwek et al. [7]. Additionally, the prodromal complaints and cortical thickening fitted the cases described by Goh et al. [5] and Kwek et al. [7].

We find it very peculiar that radiologically there seemed to be no sign of osteoporosis. The radiological examination of the femurs showed a thick cortex and small lumen of the femur, while osteoporotic bone typically shows cortical thinning and increased radiolucency. The DEXA scan in our third case did not show any sign of osteoporosis or osteopenia.

Additionally, there was no sign of fracture at the time of the periosteal reaction. This reaction is most likely due to microfractures at these high stressed points in the skeleton that produce a callus osteoid (so-called Looser's transformation).

The patients in the cases were receiving more types of medication than only the bisphosphonates. If the other medication is of any influence on the fractures remains questionable. Corticosteroids are known to cause fractures with preserved bone density. Also can corticosteroids induce apoptosis in osteoblasts and clasts thus resulting in osteoporosis and related fractures [8]. But it concerns vertebral fractures and hip fractures and no cases of subtrochanteric fractures have been described, hence the possibility of corticosteroids causing these fractures is highly improbable [9]. The same goes for proton pump inhibitors causing osteoporotic fractures [10]. But still, no cases are known with normal BMD and subtrochanteric fractures in chronic PPI use. The influence of methotrexate on bone metabolism is not known, but in our cases 1 patient did not use methotrexate at all.

In the pathology reports of the taken biopsies in patients two and three, there were no signs of hypermineralization or micro fractures. It was remarkable that there was a normal concentration of osteoclasts while bisphosphonates are assumed to inhibit osteoclasts. Also there was an imbalance between bone formation and bone resorption, resulting in increased resorption. This is in contrast with the presumption that these fractures are caused by suppressed bone turnover [11].

We have a strong suspicion but no definite evidence, backed by recent reports [12–15], that there might be a causal relation between long-term alendronate use and low-energy subtrochanteric fractures with prodromal pain. The course of the aforementioned cases seems reason enough to discontinue alendronate and to strictly follow-up the patient in order to prevent a fracture on the contra-lateral side, even, or perhaps especially, in patients using prednisone as well. Discontinuation does not significantly change the risk of non-vertebral fracture in the first 5 years. In a group of women with discontinued therapy after 5 years, the BMD remained at or above baseline values 10 years earlier and bone turnover was still somewhat reduced [16]. 

Furthermore, it should be noted that a negative DEXA scan and a thick cortex on radiological examination do not exclude the possibility of spontaneous fracture in a specific population. Additional research is necessary to determine the exact correlation between the use of alendronate and spontaneous or low-energy trauma fractures.

## 3. Conclusion

At the annual meeting of the American Academy of Orthopaedic Surgeons on the 11th of March 2008, Goh and Neviaser warned for the risks of fractures in alendronate use. They stated that further research on the negative effects of long-term alendronate use is necessary. In addition, they argued that the above mentioned fracture pattern in an area of cortical thickening could be specific to alendronate-using patients. In our opinion, the fractures seen are most appropriately named stress or fatigue fractures. In this view, patients with these prodromal symptoms should at least refrain from weight bearing of the affected limb. The alendronate should be discontinued. In case of bilateral symptoms, resulting in inability to mobilise without weight bearing, or inability to refrain from weight bearing for any other reason, preventive nailing should be considered. It remains unknown whether changed calcification results in loss of elasticity or whether the cellular response to micro-fractures in the area of the femur subjected to maximal bending stress is disturbed.
